# Differences in PPD- and mitogen-induced T-cell activation marker expression characterize immunopathology in acute tuberculosis patients

**DOI:** 10.1007/s10096-023-04741-3

**Published:** 2024-01-03

**Authors:** Isaac Acheampong, Difery Minadzi, Edwin F. Laing, Michael Frimpong, Monika M. Vivekanandan, Augustine Yeboah, Ernest Adankwah, Wilfred Aniagyei, Joseph F. Arthur, Millicent Lamptey, Mohammed K. Abass, Francis Kumbel, Francis Osei-Yeboah, Amidu Gawusu, Linda Batsa Debrah, Dorcas O. Owusu, Alexander Debrah, Ertan Mayatepek, Julia Seyfarth, Richard O. Phillips, Marc Jacobsen

**Affiliations:** 1 Kumasi Centre for Collaborative Research in Tropical Medicine (KCCR), Kumasi, Ghana; 2 Department of Medical Diagnostics, College of Health Sciences, Kwame Nkrumah University of Science and Technology (KNUST), Kumasi, Ghana; 3 School of Medicine and Dentistry, College of Health Sciences, Kwame Nkrumah University of Science and Technology KNUST, Kumasi, Ghana; 4 Agogo Presbyterian Hospital, Agogo, Ghana; 5 St. Mathias Catholic Hospital, Yeji, Ghana; 6 Atebubu District Hospital, Atebubu, Ghana; 7 Sene West Health Directorate, Kwame Danso, Ghana; 8 Department of General Pediatrics, Neonatology and Pediatric Cardiology, Medical Faculty, University Hospital Duesseldorf, Heinrich-Heine-University, 40225 Duesseldorf, Germany

**Keywords:** Tuberculosis, Immunopathology, Mitogen, T-cell activation marker, Anergy

## Abstract

Impaired T-cell responses to mitogens and high T-cell activation marker (TAM) expression on *Mycobacterium tuberculosis*–specific T-cells characterize immunopathology in patients with tuberculosis (TB). In a study of patients with TB (*n* = 60) and asymptomatic contacts (controls, *n* = 37), we found that TB patients had higher CD38^+^ T-cell proportions specific for *M. tuberculosis* protein (PPD_Mtb_), yet total proportions of PPD_Mtb_-specific T-cells were comparable. Notably, both activated (CD38^+^) and total IFN-γ^+^ T-cells from TB patients had lower mitogen (phytohemagglutinin, PHA)-induced responses. This impaired mitogen response improved the classification efficacy of the TAM-TB assay, especially employing the PPD/PHA-induced T-cell ratio.

**Supplementary Information:**

The online version contains supplementary material available at 10.1007/s10096-023-04741-3.

## Introduction

Tuberculosis remains a major health threat with approximately 10 million new cases and 2 million deaths occurring annually [1]. *M. tuberculosis* infection progresses towards acute disease in approximately 10% of index patient contacts; however, it remains asymptomatic in the vast majority of individuals due to effective immune surveillance [2]. T-helper type 1 (T_H_1) response is of central importance for host protection, but T_H_1 quantification (e.g., of the key cytokine IFN-γ) does not distinguish between patients with tuberculosis and asymptomatic *M. tuberculosis* infection [2]. However, the characterization of *M. tuberculosis*–specific T_H_1 cells by including markers of recent activation (e.g., CD38, HLA-DR) showed that this approach—termed TAM-TB—is able to identify patients with acute tuberculosis [3]. The TAM-TB assay combines in vitro stimulation of whole blood samples with *M. tuberculosis* antigens and positive control (e.g., PHA) with flow cytometry–based characterization of T-cell phenotypes.

It is well-described that immunopathology causes impaired T-cell responses in a subgroup of patients with acute tuberculosis [4, 5]. Different mechanisms identified to contribute to immune inhibition and antigen-specific as well as PHA-induced T-cells were shown to be affected in TB patients [6, 7]. Recent studies showed impaired PHA response of patients with tuberculosis in IFN-γ release assays (IGRA) and demonstrated the applicability of this marker for diagnosis of tuberculosis disease and for monitoring treatment efficacy [8–10]. In the present study, we investigated the influence of TB pathology-mediated inhibitory effects on the TAM-TB assay by comparing TB patients prior to treatment onset with asymptomatic contacts.

## Material and methods

### Study cohorts and clinical characterization

We recruited tuberculosis patients (*n* = 60) and asymptomatic contacts (controls) between April 2019 and September 2021 from the Agogo Presbyterian Hospital, St. Mathias Catholic Hospital, and Atebubu District Hospital in Ghana. Blood samples were collected before initiation of treatment. Diagnosis of tuberculosis was based on the described criteria [11]. Some of the samples from both cohorts were included in previous studies [11–13]. Controls were selected on the described criteria that showed high reliability in identifying individuals with previous *M. tuberculosis* infection caused by a respective index TB patient [8, 14]. Controls were then preselected on the basis of PPD-specific CD4^+^IFN-γ^+^ response (> 0.02%). Of the 47 tested, 37 controls fulfilled the criteria and were included in this study (Table 1).

**Table 1:** Characteristics of study participants

Parameter		TB (*n* = 60)	Controls (*n* = 47)	*P*-value
Age	(Mean ± SD)	46 ± 15.15	47.73 ± 12.96	0.5658
Gender	Male, *n* (%)	44 (73.3%)	20 (54.05%)	0.0769
	Female, *n* (%)	16 (26.7%)	17 (45.95%)	
Chest X-ray	Suggestive, *n* (%)	40 (66.7%)	NA	NA
GeneXpert	Positive, *n* (%)	53 (88.3%)	NA	NA
Smear	Positive, *n* (%)	50 (83.3%)	NA	NA
	Scanty *n* (%)	6 (12.0%)	NA	NA
	+1, *n* (%)	3 (6.0%)	NA	NA
	+2, *n* (%)	32 (64.0%)	NA	NA
	+3, *n* (%)	9 (18.0%)	NA	NA
Culture	Positive, *n* (%)	47 (78.3%)	NA	NA
Cough	> 2 weeks, *n* (%)	48 (80.0%)	NA	NA
Chest pains	*N* (%)	33 (55.0%)	NA	NA
Hemoptysis	*N* (%)	45 (75.0%)	NA	NA
Weight loss	*N* (%)	48 (80.0%)	NA	NA
Fever	*N* (%)	65 (76.7%)	NA	NA
Night sweats	*N* (%)	66 (75.0%)	NA	NA

*N* number, *NA* not applicable, *TB* tuberculosis patients

Age (*t*-test), Gender (Fisher’s exact test)

### The TAM-TB assay

The TAM-TB assay was performed as described [3]. In brief, diluted whole blood was stimulated using ESAT6/CFP10 (2 μg/ml), protein derivative of *M. tuberculosis* (PPD_Mtb_; 10 μg/ml) or PHA (10 μg/ml), as well as costimulatory antibodies (i.e, αCD28 and CD49d; 1 μg/ml each). After overnight culture in the presence of Brefeldin A, samples were stained with the following antibodies: α CD3-FITC (clone HIT3a), αCD4-PerCP-Cy5.5 (clone RPA-T4), αCD38-APC (clone HIT2), and αIFNγ-PE (clone B27), all BioLegend, and measured using a BD Acurri C6 flow cytometer. Data analyses were done using FlowJo (BD). Minimum detection was set at 0.001%. The gating strategy is illustrated in Supplementary Figure 1.

### Statistics

Non-parametric Mann–Whitney *U*-test to compare cases and contacts was performed using GraphPad Prism v9. Receiver operating characteristic (ROC) was performed to evaluate the discrimination efficacy of different parameters. A *p*-value below 0.05 was considered statistically significant.

## Results and discussion

**Fig. 1:**
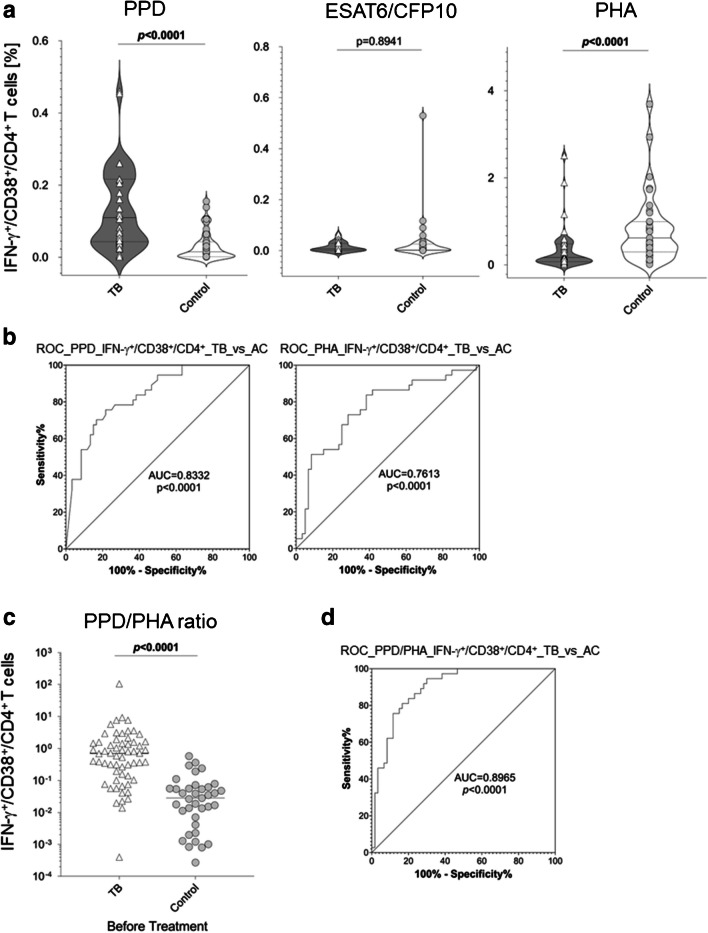
PPD_Mtb_ and PHA-induced differences in IFN-γ^+^/CD38^+^/CD4^+^ T-cells discriminate between TB patients and controls. *M. tuberculosis* antigens (i.e., PPD_Mtb_, ESAT6_CFP10) and phytohemagglutinin (PHA)-specific IFN-γ expressing CD4^+^ T-cell proportions from the in vitro culture (20 h) were measured by flow cytometry in whole blood samples from tuberculosis patients (TB, *n* = 60) and contacts (controls, *n* = 37). **a** Comparisons of PPD_Mtb_, ESAT6_CFP10 and PHA-specific IFN-γ expressing CD4^+^ T-cell proportions between the study groups of TB patients (dark grey background) and controls (open background) are shown as violin plots with stagged symbols (indicating individual donor values). A two-tailed Mann–Whitney *U*-test analysis was employed, and nominal *p*-values are shown. **b** Receiver operating characteristic (ROC) analyses for discrimination of TB patients and controls for PPD_Mtb_ and mitogen-specific IFN-γ expressing CD4^+^ T-cell proportions are shown. Area under curve (AUC) and nominal *p*-values are shown. **c** Ratio of PPD_Mtb_/PHA-induced IFN-γ^+^/CD38^+^/CD4^+^ T-cell proportions of TB (triangle symbols, open background) patients and controls (circular symbols, grey background) are shown as scattered symbol plots with lines indicating median and interquartile ranges. A two-tailed Mann–Whitney *U*-test analysis was employed, and nominal *p*-values are shown. **d** ROC analysis for discrimination of TB patients and controls for the ratio of PPD_Mtb_/PHA-induced IFN-γ^+^/CD38^+^/CD4^+^ T-cell proportions. AUC and nominal *p*-values are shown

Whole blood in vitro stimulation and flow cytometry phenotype analysis of samples from tuberculosis patients and contacts were performed using *M. tuberculosis* antigens (i.e., PPD_Mtb_, ESAT6_CFP10), and the mitogen, PHA, PPD_Mtb_, and ESAT6_CFP10-induced proportions of IFN-γ^+^ T-cell were similar between the study groups (Supplementary Figure 2). In contrast, the inclusion of CD38, a marker of recent activation, for the gating detected higher proportions of PPD-specific IFN-γ^+^/CD38^+^/CD4^+^ T-cells in patients with TB as compared to controls (*p* < 0.0001; Fig. 1a). No differences were seen for ESAT6_CFP10-specific T-cells (*p* = 0.8941; Fig. 1a). The results for PPD were in accordance with previous studies demonstrating higher proportions of recently activated *M. tuberculosis*–specific T-cells in blood samples from patients with TB [3]. Notably, significant differences were seen in the response against PHA. PHA induced lower proportions of IFN-γ^+^/CD38^+^/CD4^+^ T-cells in samples from patients with TB as compared to contacts (*p* < 0.0001; Fig. 1a). Since similar differences were seen also for all IFN-γ^+^/CD4^+^ T-cells independent of recent activation (Supplementary Figure 2), we concluded that described immunopathology effects are likely causative for reduced PHA response in patients with TB. Inflammatory pathways were shown to be associated with hyporesponsive T-cell responses in tuberculosis patients [4, 15]. Hypermethylation of DNA as well as constitutive STAT3 phosphorylation were identified as potential underlying mechanisms [7, 15]. Both, pathogen-mediated and plasma milieu effects, were identified as potential triggers [5]. In this context, a recent study found a negative correlation between high IL-6 plasma levels and impaired PHA response in TB patients [12]. No differences were detected between TB patients with high or low *M. tuberculosis* sputum burden (Supplementary Figure 3) or between female and male TB patients (Supplementary Figure 4).

Next, we applied values for PPD_Mtb_ and PHA responses to determine the discriminating capacity of these markers using receiver operating characteristic (ROC) analyses. PPD_Mtb_- and PHA-induced IFN-γ^+^/CD38^+^/CD4^+^ T-cell proportions were able to distinguish participants from both study groups with moderate efficacy (AUC, *p*-value; PPD_Mtb_, 0.83, *p* < 0.0001; PHA, 0.76, *p* < 0.0001; Fig. 1b). Previous studies indicated that a combination of both, PPD_Mtb_- and PHA-specific responses, may improve discrimination between TB patients and controls [10, 12, 16]. PPD/PHA ratios were calculated and significant differences between the study groups were detected (*p* < 0.0001; Fig. 1c). Notably, ROC-based discrimination detected a strong capacity of PPD_Mtb_/PHA T-cell response ratios to distinguish between the study groups (AUC = 0.90, *p* < 0.0001; Fig. 1d). These results confirmed the capacity of the TAM-TB assay to classify tuberculosis disease and showed the effects of immunopathology that can add to diagnosis.

### Supplementary information

ESM 1 (PDF 306 kb)

## Data Availability

The dataset generated during this study is available from the corresponding author upon reasonable request.

## References

[1] Global Tuberculosis Report (2021) Geneva: World Health Organization. 2021. Licence: CC BY-NC-SA 3.0 IGO

[2] Flynn JL, Chan J (2001). Immunology of tuberculosis. Annu Rev Immunol.

[3] Portevin D, Moukambi F, Clowes P, Bauer A, Chachage M, Ntinginya NE, Mfinanga E, Said K, Haraka F, Rachow A, Saathoff E, Mpina M, Jugheli L, Lwilla F, Marais BJ, Hoelscher M, Daubenberger C, Reither K, Geldmacher C (2014). Assessment of the novel T-cell activation marker-tuberculosis assay for diagnosis of active tuberculosis in children: a prospective proof-of-concept study. Lancet Infect Dis.

[4] DiNardo AR, Gandhi T, Heyckendorf J, Grimm SL, Rajapakshe K, Nishiguchi T, Reimann M, Kirchner HL, Kahari J, Dlamini Q, Lange C, Goldmann T, Marwitz S, Group D-Tcs, Abhimanyu, Cirillo JD, SHE K, Netea MG, van Crevel R, Mandalakas AM, Coarfa C (2022). Gene expression signatures identify biologically and clinically distinct tuberculosis endotypes. Eur Respir J.

[5] Ellner JJ (1996). Immunosuppression in tuberculosis. Infect Agents Dis.

[6] Kleinhenz ME, Ellner JJ, Spagnuolo PJ, Daniel TM (1981). Suppression of lymphocyte responses by tuberculous plasma and mycobacterial arabinogalactan. Monocyte dependence and indomethacin reversibility. J Clin Invest.

[7] DiNardo AR, Rajapakshe K, Nishiguchi T, Grimm SL, Mtetwa G, Dlamini Q, Kahari J, Mahapatra S, Kay A, Maphalala G, Mace EM, Makedonas G, Cirillo JD, Netea MG, van Crevel R, Coarfa C, Mandalakas AM (2020). DNA hypermethylation during tuberculosis dampens host immune responsiveness. J Clin Invest.

[8] Adankwah E, Lundtoft C, Guler A, Franken K, Ottenhoff THM, Mayatepek E, Owusu-Dabo E, Phillips RO, Nausch N, Jacobsen M (2019). Two-hit in vitro T-cell stimulation detects mycobacterium tuberculosis infection in QuantiFERON negative tuberculosis patients and healthy contacts from Ghana. Front Immunol.

[9] Bosco MJ, Hou H, Mao L, Wu X, Ramroop KD, Lu Y, Mao L, Zhou Y, Sun Z, Wang F (2017). The performance of the TBAg/PHA ratio in the diagnosis of active TB disease in immunocompromised patients. Int J Infect Dis.

[10] Wang F, Hou HY, Wu SJ, Zhu Q, Huang M, Yin B, Huang J, Pan YY, Mao L, Sun ZY (2016). Using the TBAg/PHA ratio in the T-SPOT((R)).TB assay to distinguish TB disease from LTBI in an endemic area. Int J Tuberc Lung Dis.

[11] Acheampong I, Minadzi D, Adankwah E, Aniagyei W, Vivekanandan MM, Yeboah A, Arthur JF, Lamptey M, Abass MK, Kumbel F, Osei-Yeboah F, Gawusu A, Laing EF, Batsa Debrah L, Owusu DO, Debrah A, Mayatepek E, Seyfarth J, Phillips RO, Jacobsen M (2023). Diminished interleukin-7 receptor expression on T-cell subsets in tuberculosis patients. Hum Immunol.

[12] Vivekanandan MM, Adankwah E, Aniagyei W, Acheampong I, Minadzi D, Yeboah A, Arthur JF, Lamptey M, Abass MK, Kumbel F, Osei-Yeboah F, Gawusu A, Debrah LB, Owusu DO, Debrah A, Mayatepek E, Seyfarth J, Phillips RO, Jacobsen M (2023). Impaired T-cell response to phytohemagglutinin (PHA) in tuberculosis patients is associated with high IL-6 plasma levels and normalizes early during anti-mycobacterial treatment. Infection.

[13] Vivekanandan MM, Adankwah E, Aniagyei W, Acheampong I, Yeboah A, Arthur JF, Lamptey MNK, Abass MK, Gawusu A, Kumbel F, Osei-Yeboah F, Debrah LB, Owusu DO, Debrah A, Mayatepek E, Seyfarth J, Phillips RO, Jacobsen M (2023). Plasma cytokine levels characterize disease pathogenesis and treatment response in tuberculosis patients. Infection.

[14] Adankwah E, Nausch N, Minadzi D, Abass MK, Franken K, Ottenhoff THM, Mayatepek E, Phillips RO, Jacobsen M (2021). Interleukin-6 and mycobacterium tuberculosis dormancy antigens improve diagnosis of tuberculosis. J Inf Secur.

[15] Harling K, Adankwah E, Guler A, Afum-Adjei Awuah A, Adu-Amoah L, Mayatepek E, Owusu-Dabo E, Nausch N, Jacobsen M (2019). Constitutive STAT3 phosphorylation and IL-6/IL-10 co-expression are associated with impaired T-cell function in tuberculosis patients. Cell Mol Immunol.

[16] Katakura S, Kobayashi N, Hashimoto H, Kamimaki C, Tanaka K, Kubo S, Nakashima K, Teranishi S, Watanabe K, Hara Y, Yamamoto M, Kudo M, Piao H, Kaneko T (2020). Identification of a novel biomarker based on lymphocyte count, albumin level, and TBAg/PHA ratio for differentiation between active and latent tuberculosis infection in Japan. Tuberculosis (Edinb).

