# Supplementary material for: Differences in PPD- and mitogen-induced T-cell activation marker expression characterize immunopathology in acute tuberculosis patients
Source: Eur J Clin Microbiol Infect Dis. 2024 Jan 3;43(3):611–6. doi: 10.1007/s10096-023-04741-3 (PMC10917863; doi:10.1007/s10096-023-04741-3)
Supplement: Supplementary file 1 — (PDF 306 kb) [file 10096_2023_4741_MOESM1_ESM.pdf]

Supplementary Figure 1

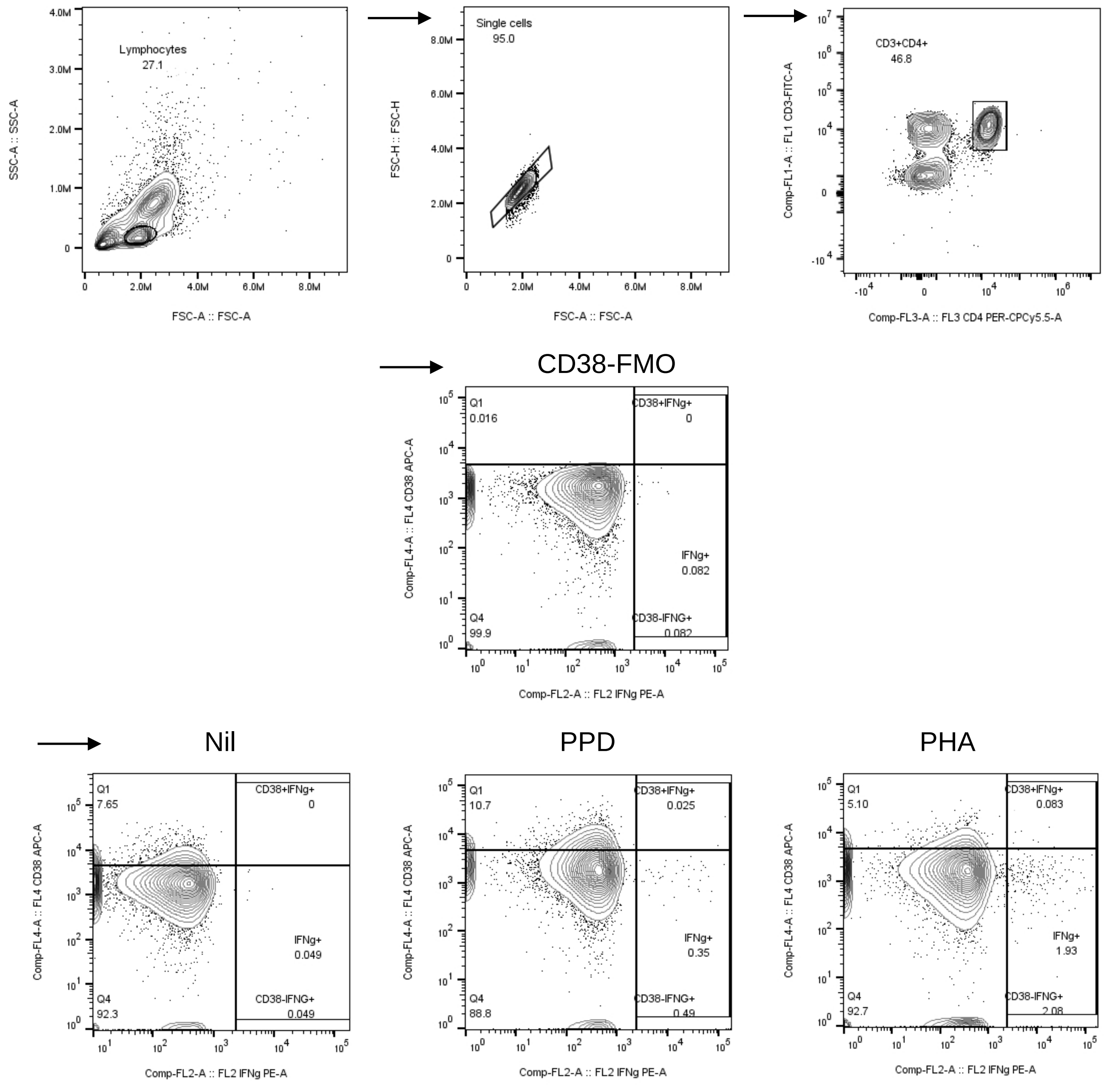

Representative depiction of the flow cytometry gating procedure. Lymphocytes were first selected based on size (forward scatter area, FSC-A) and granularity (side scatter area, SSC-A). Next, duplets were excluded using FSC-A vs. forward scatter height (FSC-H). $CD3^+$ $CD4^+$ T cells were then selected and the proportions of all IFN-γ^+ cells (gate combines quadrant (Q)2 and Q3) as well as IFN-γ^+/$CD38^+$ cells (Q2) were deduced. A fluorescence minus one (FMO) control not stained for CD38 was used to set the quadrants. Proportions of non-stimulated samples (nil) were subtracted from antigen specific and PHA-induced proportions. Contour plot with outliers are given.

Supplementary Figure 2

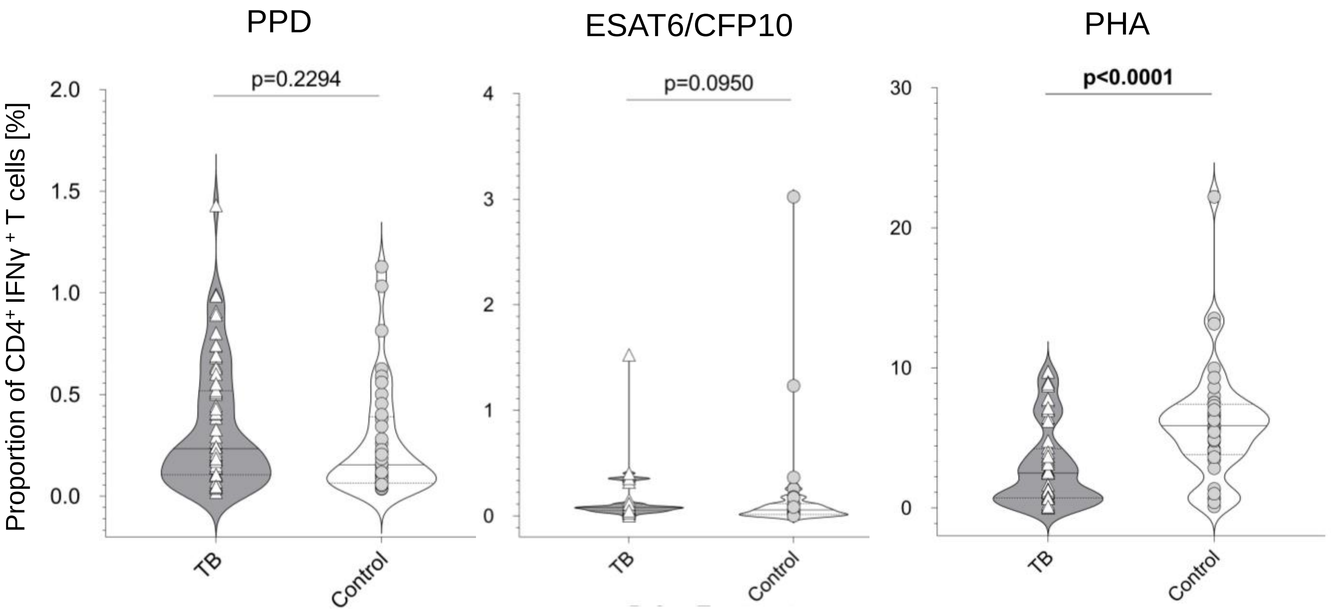

Proportions of $CD4^+$ IFNγ $^+$ T cells after *in vitro* stimulation with purified protein derivative of *M. tuberculosis* (PPD_{Mtb}), the early secretory antigen target 6/ Culture filtrate protein-10 fusion-protein (ESAT6/CFP10) and phytohemagglutine (PHA) were compared between tuberculosis patients (TB, n=60; deep grey) and controls (n=37; open background). A two-tailed Mann-Whitney U-test analysis was employed, and nominal p values are provided. Significance was achieved at $p<0.05$ and was indicated by bold letters.

Supplementary Figure 3

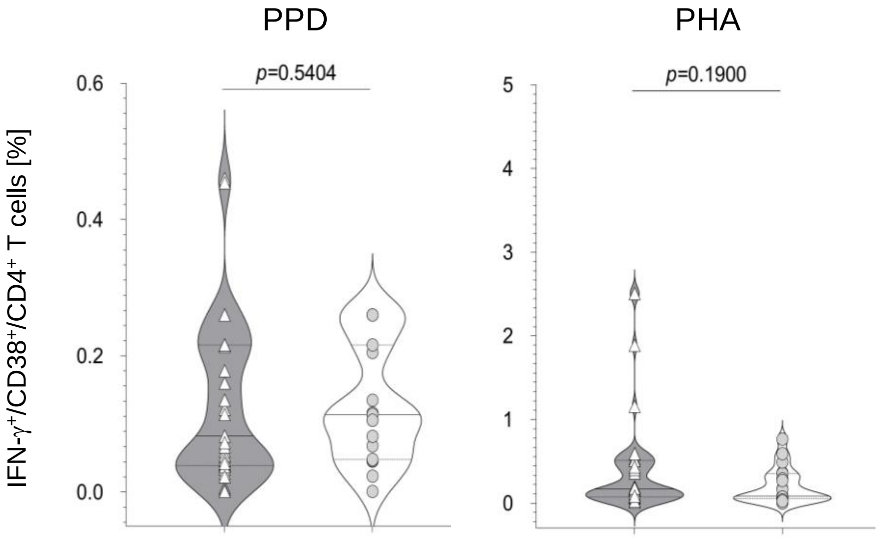

Proportions of IFN-γ^{+}/CD38^{+}/CD4^{+} T cells after *in vitro* stimulation with purified protein derivative of *M. tuberculosis* (PPD_{Mtb}) and phytohemagglutine (PHA) were compared between grade 2/3 (n=41; deep grey) and grade 1/scanty/Negative (n=19; open background) tuberculosis patients. A two-tailed Mann-Whitney U-test analysis was employed, and nominal p values are provided.

Supplementary Figure 4

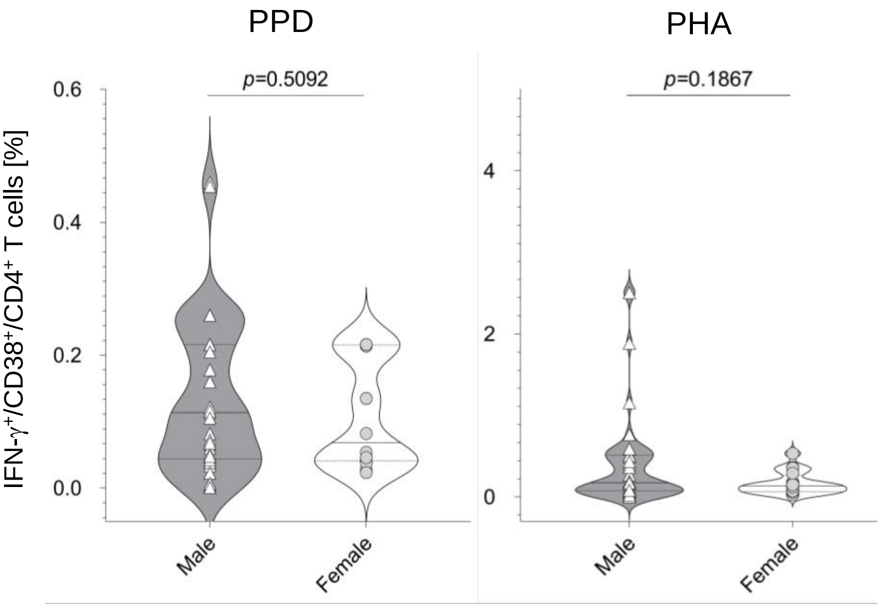

Proportions of IFN-γ^{+}/CD38^{+}/CD4^{+} T cells after *in vitro* stimulation with purified protein derivative of *M. tuberculosis* (PPD_{Mtb}) and phytohemagglutine (PHA) were compared between male (n=44; deep grey) and female (n=16; open background) tuberculosis patients. A two-tailed Mann-Whitney U-test analysis was employed, and nominal p values are provided.
